# The Effects of Ramadan Fasting and Physical Activity on Blood Hematological-Biochemical Parameters

**Published:** 2013-07

**Authors:** Seyyed Reza Attarzadeh Hosseini, Keyvan Hejazi

**Affiliations:** 1 Sport Physiology, Faculty of Physical Education and Sport Sciences, Ferdowsi University of Mashhad, Mashhad, Iran; 2 Department of Physical Education and Sports Sciences, Ferdowsi University of Mashhad, Mashhad, Iran

**Keywords:** Active and non active men, Hematological-Bioche, mical Index, Physical activity, Ramadan Fasting

## Abstract

***Objective(s)*** ***:*** Fasting during the month of Ramadan is a religious obligation and belief for healthy adult Muslims. The aim of the present study was to determine the effects of Ramadan Fasting and physical activity on ‘Blood Hematological-Biochemical Parameters’.

***Materials and Methods:*** In this study, twenty-six healthy males in two experimental groups were compared in two different instances before and after the training period. The groups which were selected by convenience sampling method were divided into two non-active fasting (n=13) and active fasting (n=13) groups. For comparison purposes between groups, paired and independent sample t-test was performed, respectively, after ensuring their normality within a significance level of *P*≤0.05.

***Results:*** HDL-C increased significantly in both active and non-active fasting groups, Moreover, amount of hematocrit (Hct), red blood cell count (RBC), TC, LDL, VLDL, LDL/HDL and TC/ HDL decreased significantly. Amount of hemoglobin (Hgb) and glucose reduced significantly in the active-fasting group. The variation of the means between the groups in the Hgb index and LDL/HDL were statistically significant.

***Conclusion:*** Fasting during the month of Ramadan by regular physical activity caused positive alterations in Hematological-Biochemical Index. These changes may be due to the alterations in diet, biology response of the body to the starving and physical activity during this month.

## Introduction

Ramadan is considered as one of the five pillars of Islam which is followed by about 400 million Muslims worldwide (1). During the month of Ramadan, Muslims abstain from intake of food and drink from sunrise to sunset. Mainly, food products such as fluids and food are used at night; and the number of meals, sleep quantity, and physical activity are reduced at this month. Dietary habits during Ramadan is not similar to the rest of the year, therefore, the amount of consumed fat, protein and carbohydrate may vary during this month (2). The fasting period may vary from 10 to 19 hours per day (3).

Previous investigations have examined the effect of Ramadan fasting and physical activity on hematological-biochemical responses during ritual fasting such as Ramadan (-). In fact, hematocrit and hemoglobin have both been reported to increase (7), decrease (8), and without any changes (9) during the month of Ramadan.

Ramadan fasting has been demonstrated to change the lipoid profile of athletes. TC, HDL-C and LDL-C have been shown to elevate in elite judokas (10). In addition, free fatty acid levels have been shown to increase in middle-distance runners (11). 

Due to the fact that blood lipid levels can change considerably during the month of Ramadan, In this regard, the lipid profile should be monitored regularly during the months.

However, it is not clear which reason has the major effect on lipid profile. Conflicting results have been reported that the total cholesterol during weight loss and/ or Ramadan fasting decreases (12, 13); without any changes (14, 15) and even increases (16). Adlouni *et al *(17) reported that fasting during Ramadan led to a significant decrease in a serum total cholesterol, triglyceride and LDL-C, while a significant increase in the serum HDL-cholesterol during this month. On the other hand, Maislos *et al* (18) noted that LDL-C, very-low-density lipoprotein (VLDL) and total-cholesterol have not changed, while they observed a significant increase in HDL-cholesterol levels and a reduce in the ratios LDL/HDL and TC/LDL at the end of Ramadan. There is a controversy in research results: Sarraf-Zadegan *et al *(19) and Argani *et al* (15) showed no changes in WBC count or another hematological parameters throughout Ramadan; Bouhlel *et al* (7) Showed a significant increase in Hb and Hct in rugby players in Tunisia. However, Argani et al.(15) Showed no changes in Hb in renal transplant recipients during Ramadan.

The conflicting results about the effects of fasting on biochemical parameters and hematological serum in previous studies along with the lack of sufficient evidence in examining the effects of sports activities in the holy month of Ramadan and the increase of fasting time in summer time have given great value to an investigation of fasting with or without regular sports activities. The significance of fasting, especially fasting along with sports activities, and also better understanding of physiological conditions of athletes during the holy month of Ramadan have made researchers conducting some comparison studies on the effect of Ramadan and regular sports activities on biochemical and hematological serums of male athletes (soccer players) and male non-athletes (passive). 

## Materials and Methods

***Subjects***

This research was semi-experimental with two phases which was performed before and after the Ramadan fasting in the experimental and the control groups. During the first stage, written informed consent was obtained from all the 26 young active and non-active males in Ramadan, 2012. All participants were asked to fill out a medical history as well as a medical questionnaire to ensure that they were not taking any regular medications for conditions such as: cardiovascular, respiratory, renal and metabolic diseases. Also, all subjects were completely familiarized with all of the experimental procedures and exercise protocol (20). The volunteers were assigned randomly into one of two groups as follows: a control group (n=13), training group (n=13). 

During the second stage, their heights were measured in centimeters using a height determiner and their weight was recorded using a digital scale produced by a German company called Beurer (PS07-PS06). Then, the waist-hip ratio was determined. Body fat percentage was calculated using a body compound determiner (model In-body-720 made in Korea) and based on a method called bioelectrical impedance. All of these measurements were carried out while the runners had stopped eating or drinking four hours prior to their test, and their bladder, stomach, and bowels were empty.

***Exercise Programs***

The football training session was started in the evening of the first day till 30th day of Ramadan (15 to 16:30 pm). It was included 3 sessions per week, and each session lasted for 90 minutes. In this study, regular exercise was an exercise program specifically in male elite soccer players during Ramadan. The exercise protocol included: 10 min general warm-up (walking, stretching and movement exercise); 10 min special warm-up (start short and fast movements with the ball and 10 speed starts 10 to 15 m). Then, 45 to 60 min specific training consists of: technical training, knocking, and dribbling and playing in small groups (3 × 3, 4 × 4, 5 × 5 and 6 × 6) to the alternative with intensity of 60-75 percent of maximum heart rate reserve (MHRR), according to the Table 1. At the end of each exercise session, for 10 minutes to return the body to its normal, activities such as jogging, walking and stretching was performed (21). 

According to the MHRR, variables for each athlete were calculated based on Karvonen equation (1) and was controlled during an exercise by a heart rate monitor (made in Finland–Polar).

Equation (1): Target heart rate= [%60 or %70× [((220- age)- Resting heart rate]] + Resting heart rate

In this research, we have estimated the consumed oxygen of subjects using Cooper Fitness Test (12 minutes of running), and by controlling the heartbeat cycle, using a polar heartbeat counter along with estimating the activity oxygen consumption. We could also count the partial consumed calories in each exercise session, using the indirect calorie counting. In this research, soccer players consumed 600 up to 1000 kilo calories in each exercise session.

***Biochemical tests***

Blood samples in all related studies were collected by venepunction from forearm vein after at least 15 minutes of sitting at rest or in the supine position. Blood sample was poured into a tube containing K2EDTA and mixed for 15 min before the analysis. This was performed after centrifusing samples in plastic capillary tubes using Haemato Spin Centrifuge device (Hct, Hawksly, Susses, UK). Serum biochemical concentrations were determined using an auto-analyzer spectrophotometer and different kits in various wavelengths as follows below. Serum cholesterol concentration was determined as mg/dl by using Pars-Azmun kits with chod-pap method at 546 nm wavelengths. Serum glucose concentration was determined as mg/dl by using Pars-Azmun kits with Godpap method at 500-546 nm wavelengths. Serum triglycerides concentration was determined as mg/dl by using Pars-Azmun kits with Gpopap enzymatic method at 546 nm wavelengths. High density lipoprotein (HDL) and low density lipoprotein (LDL) were measured by the enzymatic method technique, using Man kit, Tehran-Iran. Hemoglobin and hematocrit concentration were analyzed by system K-4500 automated hematology analyzer.

**Table 1:** Classification of exercise intensity

The relative intensity (percent) *			
Classification of exercise intensity	The rate of perceived exertion	Heart rate reserve	Maximum heart rate
Very light	<9	<30	<35
Moderate	10-11	30-49	35-59
Average	12-13	50-74	60-79
Heavy	14-16	75-84	80-89
Very heavy	>16	≥85	≥90

* Data from the Pollock and Wilmore (1990) (18)

***Statistical analysis***

All statistical analyses were performed with SPSS version 11.5. The average and standard deviation of data were calculated after checking the noremal distribution using Kolmogorov-Smirnov test and Homogeneity of variance method and then examined by comparison of means within and between means groups Paired-Samples t-test and Independent t-test was used respectively. Statistical significance was assigned at *P* < 0.05 for all analysis.

## Results

The mean and standard deviation of age in experimental and control group were 19.38 ± 0.5 and 21.07±1.55 years, respectively. Results of the effect of Ramadan fasting on plasma lipids are shown in Table 2. A significant reduction of serum TC, LDL, LDL/HDL, 

TC/HDL and VLDL values was observed after Ramadan fasting compared to that of before Ramadan in both groups. While, a significant reduction of serum FBS was noted only in the experimental group (*P*=0.000). A significant increase in HDL–C was observed in both groups (*P*=0.023 and *P* =0.042). No significant changes were observed on triglycerides and TG/HDL in both groups.

No significant changes were found in white blood cells, Platelets in both groups. . However, there was a significant reduction of red blood cell count in both groups, while, haemoglobin and hematocrit values decreased significantly after Ramadan compared to before Ramadan (*P* =0.027, p=0.031 respectively) in the experimental group. Hematocrit values were increased significantly after Ramadan compared to before Ramadan (*P* = 0.042) in the control group as illustrated in Table 3.

## Discussion

During the month of Ramadan, Muslims worldwide are obliged to fast during daytime hours, and restricted food and drink intake after the sunset. People may alter their sleeping habits and stay awake most of the night. Previous studies have reported conflicting results regarding the effect of Ramadan fasting on various hematological indices. 

In the present study, no significant changes were observed in white blood cell count, platelets in both groups. However, a significant reduction of red blood cell count was observed in both groups, while, haemoglobin and hematocrit values decreased significantly after Ramadan compared to before Ramadan, which was consistent with others (8, 22). In another study, Dewanti *et al* showed a significant decrease in haemoglobin and Hematocrit. Maughan *et al* (8) noted a decrease in both parameters in soccer players. While, other investigations have reported opposing findings, (7, 9, 10, 23). Chaouachi *et al* (10) noted an increase in hematocrit and a decrease in hemoglobin in elite judokas, Tayebi *et al *(9) showed no changes in hematocrit and hemoglobin. Disparity in the results of the aforementioned studies may be due to temperature, climate, fluid intake and exercise regime.

**Table 2:** The levels of some biochemical variables measured in subjects before and after Ramadan (Mean±SD)*

Variables	Groups	Pre-test Mean±SD*	Post-test Mean±SD*	P*	Different between pre-post test	P***
FBS (mg/dl)	Exercise group	91.15±5.61	85.15±5.01	†0.00	6	0.440
	Control group	88.38±6.30	84.46±7.55	0.05	3.92	
TG (mg/dl)	Exercise group	51.92±14.21	51.92±13.08	1.00	0.00	0.247
	Control group	97.15±57.46	107.30±52.45	0.209	-10.15	
TC (mg/dl)	Exercise group	145.23±9.81	140.23±9.22	† 0.002	5.00	0.223
	Control group	185.46±37.13	175.84±33.90	† 0.015	9.62	
LDL (mg/dl)	Exercise group	93.69±8.75	89.92±5.96	† 0.022	3.77	0.056
	Control group	128.07±30.52	117.00±27.31	† 0.006	11.07	
HDL mg/dl)	Exercise group	41.23±6.31	43.30±5.66	†0.023	-2.07	0.248
	Control group	37.92±3.98	38.92±3.70	† 0.042	-1	
LDL/HDL (mg/dl)	Exercise group	2.31±0.38	2.10±0.25	† 0.006	0.21	† 0.006
	Control group	3.43±0.96	3.43±0.96	† 0.004	0.00	
TG/HDL (mg/dl)	Exercise group	1.29±0.40	1.23±0.41	0.527	0.06	0.266
	Control group	2.62±1.65	2.82±1.50	0.352	-0.2	
TC/HDL (mg/dl)	Exercise group	3.57±0.41	3.26±0.30	†0.001	0.31	0.525
	Control group	4.96±1.20	4.56±0.98	†0.005	0.4	
VLDL (mg/dl)	Exercise group	10.38±1.44	9.10±1.44	†0.045	1.28	0.629
	Control group	19.43±11.49	18.49±10.85	†0.043	0.94	

*Data presented as mean ± standard deviation ** Paired sample t-test

‡The mean difference is significant at the 0.05 level *** Independent samples t-test

**Table 3:** Values of hematological indices in subjects before and after Ramadan (Mean±SD)*

Variables	Groups	Pre-test Mean±SD*	Post-test Mean±SD*	P*	Different between pre-post test	P***
White blood cell count (x 106/mm3)	Exercise group	5.66±1.75	5.21±1.00	0.252	0.45	0.259
	Control group	5.97±0.97	6.03±1.02	0.813	-0.06	
Red blood cell count (x 106/mm3)	Exercise group	5.03±0.28	4.93±0.27	0.040†	0.1	0.705
	Control group	4.98±0.26	4.86±0.24	0.032†	0.12	
Hemoglobin (gm/dl)	Exercise group	14.55±1.19	14.23±1.01	0.027†	0.32	0.031†
	Control group	14.80±1.15	14.93±1.06	0.413	-0.13	
Hematocrit (%)	Exercise group	42.19±2.81	41.45±2.43	0.031†	0.74	0.769
	Control group	42.83±2.58	41.95±2.23	0.042†	0.88	
Platelets (1000)	Exercise group	202.92±39.64	203.46±44.74	0.954	-0.54	0.809
	Control group	206.23±51.35	209.23±53.28	0.497	-3	

*Data presented as mean ± standard deviation           ** Paired sample t-test

‡The mean difference is significant at the 0.05 level.    *** Independent samples t-test

Ramadan fasting had no effect on blood platelets. This finding is contrary to the results of Ramadan *et- al* (24) Who exhibited a decrease in blood platelets by a deficiency of iron and vitamins during the month of Ramadan.

In our study, mean plasma glucose showed a significant reduction after Ramadan in comparison to initial levels. Other studies have also reported marked decline in the glucose level during Ramadan (17, 25, 26). This finding strongly indicates the beneficial effects of Islamic fasting in diminishing plasma glucose. In prolonged hunger, plasma glucose concentration decreases to the lowest possible level and after one week of hunger it starts to increase. If hunger continues for more than three weeks, glucose exceeds the initial levels. In ordinary conditions, glycogenolysis maintains plasma glucose levels around normal values for 12-16 hours and in prolonged hunger this phenomenon is able to preserve plasma glucose levels in normal ranges for 2-10 d and then after, breaking-down of fats is the most important factor interfering with decrease in glucose. In this situation, the role of the important parameters interfering with the decrease in the level of glucose is shown. In this situation, the role of Insulin and Glucagon is prominent and essential (27).

The effect of Ramadan fasting on lipid profile is different in published articles and this may be due to changes in dietary regime, decreased activity and some 

cultural parameters. Our study showed a significant increase in HDL at the end of Ramadan. Similar to other series of studies (10, 28, 29) a significant decrease was observed in LDL, total cholesterol, VLDL, LDL/HDL, TC/HDL in both groups. However, mechanisms that examine the effect of fasting on increasing the level of HDL-C are not well understood, Probably, weight loss in fasters during Ramadan may lead to an increase in the level of HDL-C. A decrease in the total cholesterol, LDL and an increase in HDL were observed in hyperlipidemic fasted subjects. Decline in lipid-related risk factors has been observed among people who are using low-calorie diet during fasting (30, 31). Future studies should include the measurement of other apolipoproteins for estimation of lipids profile of subjects. 

## Conclusion

It seems that more researches are required for evaluating the effects of Ramadan fasting and physical activities and there are yet many unanswered questions in this relation. We believe that repeated fasting, results in adaptations which result in an increased reliance upon lipid oxidation at rest and during exercise, and an increased capacity of skeletal muscle and liver to store carbohydrate. Fasting during Ramadan may be used as a good human model for dietary. There is stronger evidence for those who are fasting during Ramadan who are able to promote the ability of the body to use the lipids efficiently at rest and during exercise. If an individual can participate in exercise during Ramadan, these adaptations are more pronounced.
